# Predictive accuracy of risk factors and markers: a simulation study of the effect of novel markers on different performance measures for logistic regression models

**DOI:** 10.1002/sim.5598

**Published:** 2012-09-10

**Authors:** Peter C Austin, Ewout W Steyerberg

**Affiliations:** a Institute for Clinical Evaluative SciencesToronto, ON, Canada; b Department of Health Management, Policy and Evaluation, University of TorontoToronto, ON, Canada; c Dalla Lana School of Public Health, University of TorontoToronto, ON, Canada; d Department of Public Health, Erasmus Medical CentreThe Netherlands

**Keywords:** logistic regression, predictive model, predictive accuracy, Brier score, discrimination, c-statistic, ROC curve

## Abstract

The change in c-statistic is frequently used to summarize the change in predictive accuracy when a novel risk factor is added to an existing logistic regression model. We explored the relationship between the absolute change in the c-statistic, Brier score, generalized *R*^2^, and the discrimination slope when a risk factor was added to an existing model in an extensive set of Monte Carlo simulations. The increase in model accuracy due to the inclusion of a novel marker was proportional to both the prevalence of the marker and to the odds ratio relating the marker to the outcome but inversely proportional to the accuracy of the logistic regression model with the marker omitted. We observed greater improvements in model accuracy when the novel risk factor or marker was uncorrelated with the existing predictor variable compared with when the risk factor has a positive correlation with the existing predictor variable. We illustrated these findings by using a study on mortality prediction in patients hospitalized with heart failure. In conclusion, the increase in predictive accuracy by adding a marker should be considered in the context of the accuracy of the initial model. Copyright © 2012 John Wiley & Sons, Ltd.

## 1 Introduction

Logistic regression models are frequently used for developing prediction models for dichotomous outcomes. Examples include the Enhanced Feedback for Effective Cardiac Treatment-Heart Failure (EFFECT-HF) mortality prediction model for predicting mortality in patients hospitalized with heart failure 1 or the Global Utilization of Streptokinase and Tissue Plasminogen Activator for Occluded Coronary Arteries (GUSTO) trial (GUSTO-I) model for predicting short-term mortality in patients hospitalized with an acute myocardial infarction 2. Clinical prediction models can be developed for several reasons: they permit effective risk stratification of patients, they can be used in the design and analysis of randomized controlled trials 3, and they can be used for risk adjustment purposes when comparing patient outcomes between healthcare providers 4.

Authors have proposed several different measures to describe the predictive accuracy of a logistic regression model. These include the c-statistic (equivalent to the area under the receiver operating characteristic curve, often denoted by AUC), Brier score, Nagelkerke's generalized *R*^2^, and the discrimination slope 5–7.

The objective to the current paper was to explore changes in the c-statistic, Brier score, generalized *R*^2^, and the discrimination slope in situations in which a risk factor or novel marker is added to an existing model. To address this objective, we performed an extensive set of simulations reflecting scenarios that reflect current epidemiological research on new risk factors. We illustrated our findings using a dataset in which we examined whether patient frailty provided prognostically important information beyond that provided by simple mortality prediction models for patients hospitalized with heart failure.

## 2 Methods: measures of predictive accuracy considered

The c-statistic is the rank order statistic for predictions against true outcomes. It is calculated by taking all possible pairs of subjects consisting of one subject who experienced the outcome of interest and one subject who did not experience the outcome. The c-statistic is the proportion of such pairs in which the subject who experienced the outcome had a higher predicted probability of experiencing the event than the subject who did not experience the outcome 7. Reporting the model c-statistic is an essential component of assessing the predictive accuracy of a logistic regression model 5 but has been criticized as being insensitive to adding information from forthcoming genetic markers and biomarkers 8.

Brier score, the mean squared prediction error, is defined as 
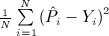
, where *N* denotes the sample size, 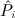 is the predicted probability of the outcome, and *Y*_*i*_ is the observed outcome (1/0). We also considered the scaled Brier score, in which Brier score is scaled by its maximum possible score: Brier_scaled_ = 1 − Brier/Brier_max_, where Brier_max_ = mean(*p*) × (1 − mean(*p*))^2^ + (1 − mean(*p*)) × mean(*p*)^2^, with mean(*p*) denoting the average probability of the outcome 4. The generalized 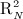 index is defined by 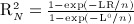, where LR is the global log likelihood ratio statistic for testing the importance of all *p* predictors in the regression model and L^0^ is the − 2 log likelihood for the null model 7,9,10.

Finally, Pencina *et al.* have suggested that the improvement in prediction due to novel risk factors be summarized using the Integrated Discrimination Improvement (IDI) 2008. Given a new regression model that includes a novel risk factor and an older regression model in which this risk factor is omitted, the IDI is estimated as follows: 

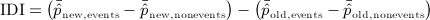
 where 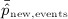 is the mean of the new model-based predicted probabilities of an event for those who develop events, whereas 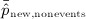 is the mean of the new model-based predicted probabilities of an event for those who do not develop an event. 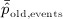 and 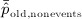 are defined similarly for the old regression model. The term in the first parenthesis is the discrimination slope for the new model, whereas the term in the second parenthesis is the discrimination slope for the old model. It has been noted that the IDI is related to the change in the proportion of variance explained between the model with and without the novel marker or risk factor 11.

We note that (scaled) Brier and IDI consider the squared distances between predicted probabilities and observed outcomes. Nagelkerke's *R*^2^ is related to the LR statistic and considers these distances on a logarithmic scale 4.

The c-statistic, Brier score, and the generalized *R*^2^ can be estimated using the val.prob function in the Design package for R. The IDI can be estimated using the improveProb function in the Hmisc package for R.

## 3 Methods: Monte Carlo simulations

In this section, we describe a series of Monte Carlo simulations that were conducted to examine the incremental increase in model accuracy due to the inclusion of a risk factor or novel marker in a logistic regression model. We considered settings in which the risk factor or marker was dichotomous and settings in which it was continuous. We also allowed the correlation between the risk factor and a continuous predictor variable to vary across the simulations.

We randomly simulated two predictor variables for each of 1000 subjects. We simulated the first predictor variable from a normally distributed random variable with mean 0 and standard deviation *σ*_c_. When the second covariate (the risk factor) was dichotomous, it was simulated from a Bernoulli distribution with parameter *p*_binary_; when it was continuous, it was simulated from a standard normal distribution. We determined a linear predictor as follows: logit(*p*_*i*_) = − 1 + *β*_*c*_*x*_*c*,*i*_ + *β*_*rf*_*x*_*rf*,*i*_, where *p*_*i*_ denotes the subject-specific probability of the binary outcome occurring and *x*_*rf*_ denotes the dichotomous or continuous risk factor. For each subject, we then randomly generated a binary outcome from a Bernoulli distribution with subject-specific parameter *p*_*i*_. We then fit two logistic regression models in the simulated dataset. The first was a univariate logistic regression model in which the binary outcome was regressed on the continuous predictor variable *X*_*c*_. The second was a logistic regression model that included both the continuous variable *X*_*c*_ and the risk factor *X*_*rf*_. We estimated each of the measures of predictive accuracy discussed in Section 2 using each of the logistic regression models. We calculated the difference in each measure of accuracy between the two logistic regression models. As noted previously, the difference between the two discrimination slopes is the IDI. We repeated this process 1000 times.

This set of Monte Carlo simulations used a full factorial design. When the risk factor was dichotomous, the following factors were varied: exp(*β*_*c*_) (the odds ratio relating the independent continuous predictor variable to the binary outcome), exp(*β*_*rf*_) (the odds ratio relating the dichotomous risk factor to the binary outcome), *σ*_c_ (the standard deviation of the independent continuous predictor variable), and *p*_binary_ (the prevalence of the binary predictor variable). We allowed exp(*β*_*c*_) to vary from 1 to 4 in increments of 0.25, *σ*_c_ to vary from 0.25 to 4 in increments of 0.25, exp(*β*_*rf*_) to take on the values 1.25, 1.5, 2, 3, and 5, and *p*_binary_ to take on the values 0.1, 0.25, and 0.5. We thus considered 3120 different scenarios when the risk factor was dichotomous. When the risk factor was continuous, the following factors were varied: exp(*β*_*c*_) (the odds ratio relating the independent continuous predictor variable to the binary outcome), exp(*β*_*rf*_) (the odds ratio relating the continuous risk factor to the binary outcome), and *σ*_c_ (the standard deviation of the independent continuous predictor variable). We allowed exp(*β*_*c*_), *σ*_c_, and exp(*β*_*rf*_) to vary as above. We thus considered 1040 different scenarios when the risk factor was continuous. In each of these scenarios, we computed the mean improvement in predictive accuracy due to the inclusion of the risk factor across the 1000 simulated datasets.

In the above two scenarios, it was assumed that the binary or continuous risk factor was independent of the continuous covariate that was included in the logistic regression model. We modified the above two scenarios by inducing a correlation between the continuous covariate and the risk factor. When the risk factor was continuous, the continuous covariate and the risk factor was simulated from the following multivariate normal distribution: 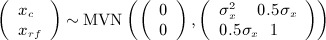, where the second matrix denotes the variance–covariance matrix of the bivariate normal distribution. Thus, the correlation between the two continuous variables was 0.5. When the risk factor was dichotomous, we simulated the continuous covariate and a second continuous covariate from the following multivariate normal distribution: 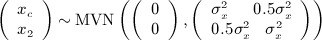 (the second continuous covariate can be thought of as a latent variable underlying the dichotomous risk factor). The correlation between the continuous covariate and the continuous latent variable will be 0.5. We dichotomized the second continuous covariate, so that the risk factor was defined to be present if the second continuous exceeded a specified threshold. We chose this threshold so that the risk factor was present for the desired proportion of subjects (*p*_binary_). We then modified the above scenario to examine a setting in which the correlation between the continuous predictor variable and the risk factor was 0.8. We thus examined settings with a moderate and strong correlation between the continuous predictor variable and the risk factor.

In the aforementioned six sets of scenarios, we examined the effect of adding a dichotomous or continuous risk factor to a logistic regression model that contained a single continuous covariate. We examined a seventh set of scenarios in which the existing logistic regression model consisted of two independent covariates: a normally distributed covariate and a dichotomous covariate that followed a Bernoulli distribution with parameter 0.5. We restricted our attention to settings in which the risk factor was dichotomous. The following factors were varied: exp(*β*_*c*_) (the odds ratio relating the independent continuous predictor variable to the binary outcome), exp(*β*_*rf*_) (the odds ratio relating the dichotomous risk factor to the binary outcome), *σ*_c_ (the standard deviation of the independent continuous predictor variable), and *p*_binary_ (the prevalence of the dichotomous risk factor). We allowed exp(*β*_*c*_) to vary from 1 to 4 in increments of 0.25, *σ*_c_ to vary from 0.25 to 4 in increments of 0.25, exp(*β*_*rf*_) to take on the values 1.25, 1.5, 2, 3, and 5, and *p*_binary_ to take on the values 0.1, 0.25, and 0.5. We assumed that the odds ratio relating the binary covariate to the outcome was equal to half of the odds ratio relating the continuous covariate to the outcome. We thus examined 3120 scenarios in which the initial logistic regression model had two covariates.

For all the simulations, we randomly generated data using the R statistical programming language 12.

## 4 Results: Monte Carlo simulations

### 4.1 Binary risk factor

**Figure 1:**
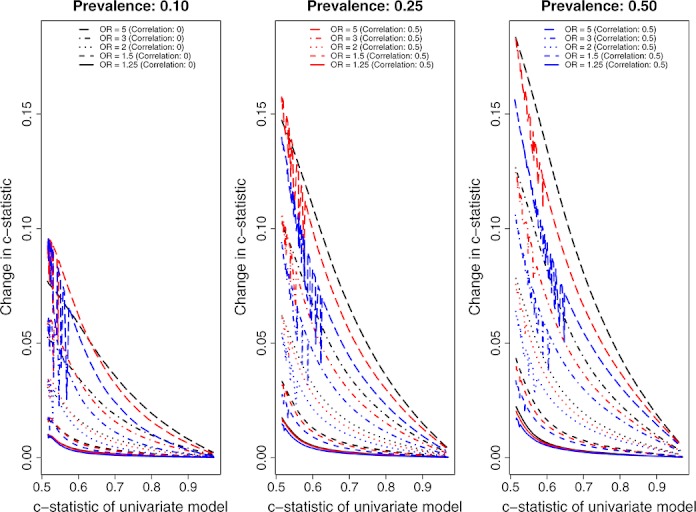
Relationship between change in c-statistic and c-statistic of univariate model (binary risk factor).

**Figure 2:**
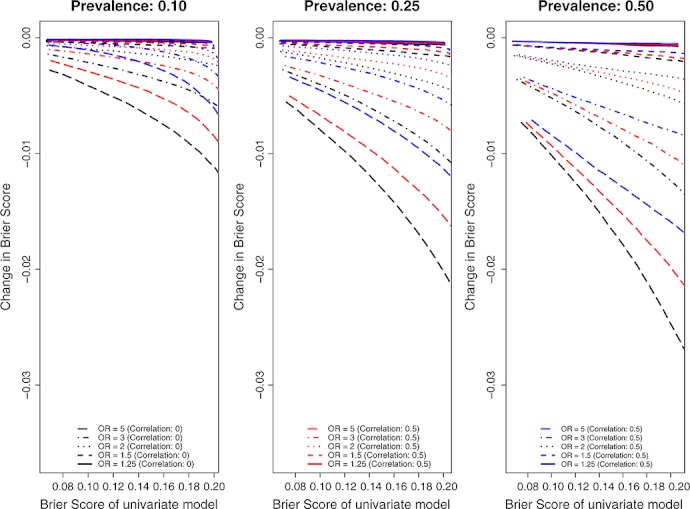
Relationship between change in Brier score and Brier score of univariate model (binary risk factor).

**Figure 3:**
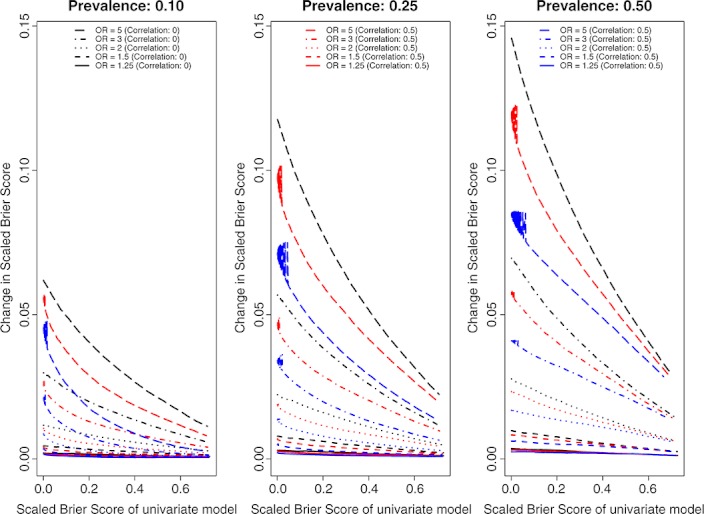
Relationship between change in scaled Brier score and scaled Brier score of univariate model (binary risk factor).

**Figure 4:**
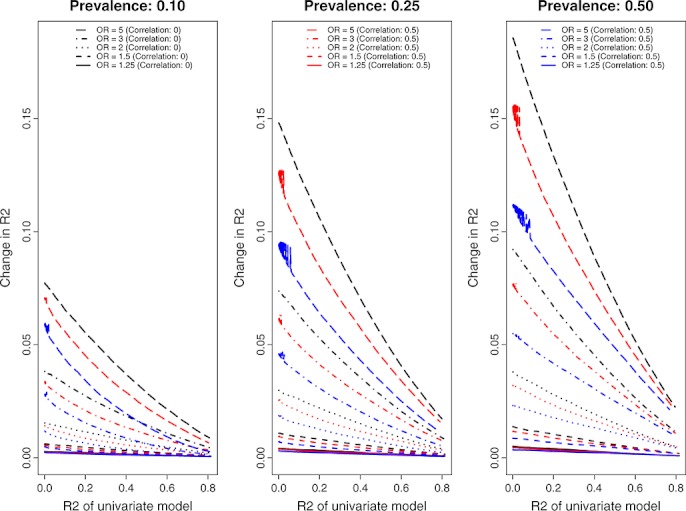
Relationship between change in *R*^2^ and *R*^2^ of univariate model (binary risk factor).

**Figure 5:**
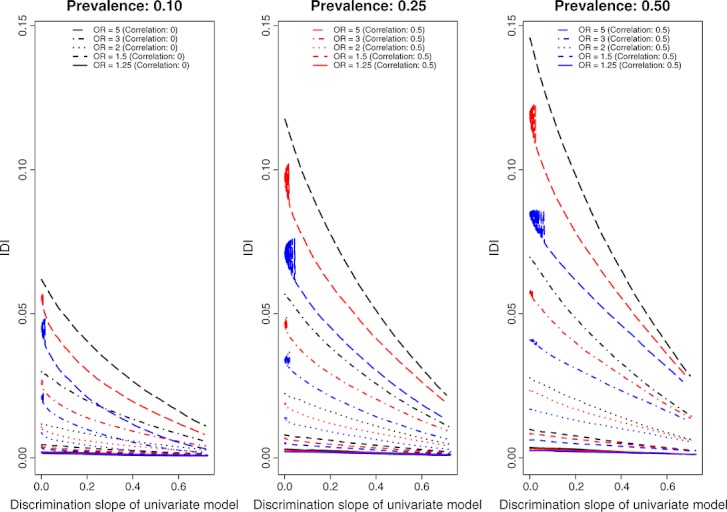
Relationship between Integrated Discrimination Improvement (IDI) and discrimination slope of univariate model (binary risk factor).

We describe the relationship between the mean change in predictive accuracy due to the inclusion of a dichotomous risk factor and the predictive accuracy of the univariate logistic regression model across the 3120 scenarios in Figures 1–5. Note that lower values of Brier score are indicative of greater predictive accuracy, whereas for remaining measures of model accuracy, greater values are indicative of greater predictive accuracy. Several observations warrant mention. First, for each combination of odds ratio and prevalence of the binary risk factor, the improvement in predictive accuracy due to the inclusion of the binary risk factor was inversely proportional to the predictive accuracy of the model that included only the continuous predictor variable. Second, when the prevalence of the binary risk factor and the predictive accuracy of the univariate model were fixed, the change in predictive accuracy was proportional to the odds ratio for the binary risk factor. Third, holding the other factors fixed, the improvement in predictive accuracy was proportional to the prevalence of the binary risk factor: we observed greater improvements in predictive accuracy as the prevalence of the binary risk factor increased. Finally, for a given measure of model accuracy, the improvement in model accuracy due to the inclusion of the binary risk factor tended to decrease as the correlation between the risk factor and the continuous predictor variable increased. The greatest increase in model accuracy tended to be observed when the risk factor and the predictor variable were uncorrelated with one another. The magnitude of the effect of correlation tended to increase as the odds ratio for the binary risk factor increased.

### 4.2 Continuous risk factor

**Figure 6:**
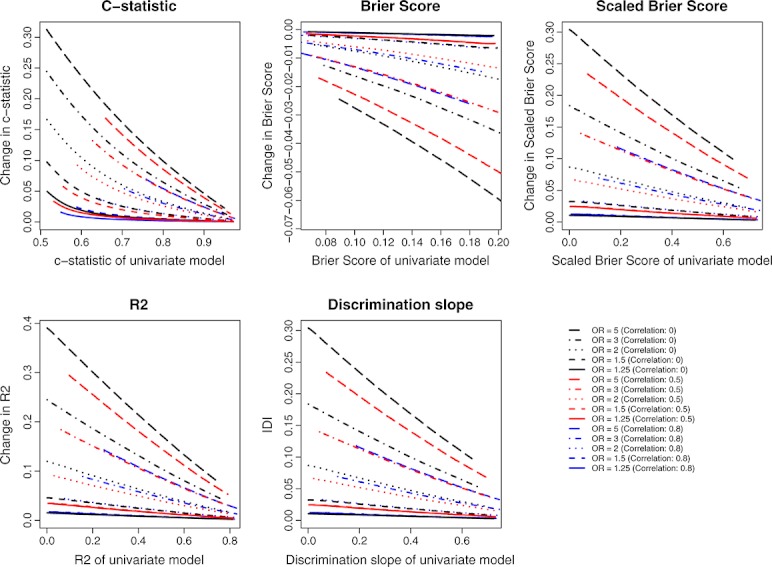
Continuous risk factor.

We describe the relationship between the mean change in predictive accuracy due to the inclusion of a continuous risk factor and the predictive accuracy of the univariate logistic regression model across the 1040 scenarios in Figure 6. As mentioned previously, several observations warrant mention. First, for each odds ratio relating the continuous risk factor to the binary outcome, the improvement in predictive accuracy due to the inclusion of the continuous risk factor was inversely proportional to the predictive accuracy of the model that included only the continuous predictor variable. Second, when the predictive accuracy of the univariate model was fixed, the change in predictive accuracy was proportional to the odds ratio for the continuous risk factor. Finally, for a given measure of model accuracy, the improvement in model accuracy due to the inclusion of the continuous risk factor decreased as the correlation between the risk factor and the continuous predictor variable increased. We observed the greatest increase in model accuracy when the risk factor and the predictor variable were uncorrelated with one another.

### 4.3 Multivariable model

**Figure 7:**
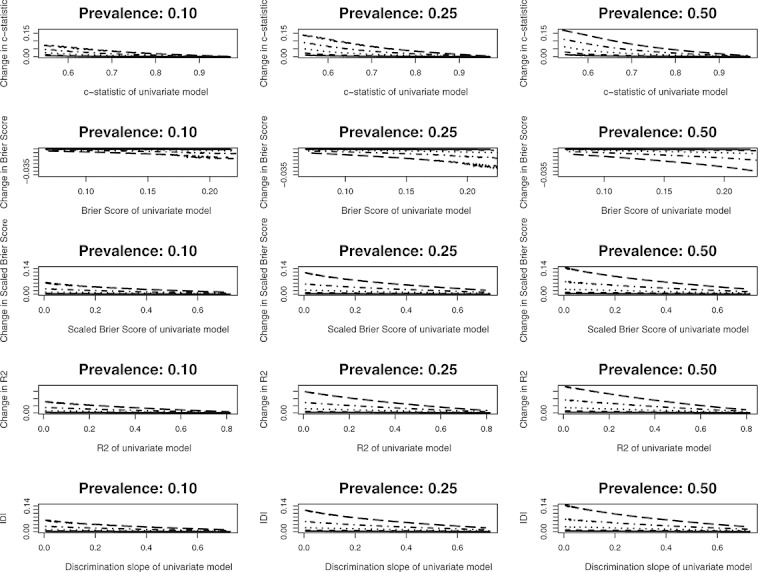
Multivariable risk model.

In the final set of scenarios, we examined the improvements in model accuracy due to the addition of a binary risk factor to a logistic regression model that contained two predictor variables (a normally distributed variable and a Bernoulli-distributed variable). We describe the results in Figure 7. We observed findings similar to the settings in which a binary risk factor was added to a logistic regression model containing a single continuous predictor variable.

### 4.4 Miscellaneous analyses

**Figure 8:**
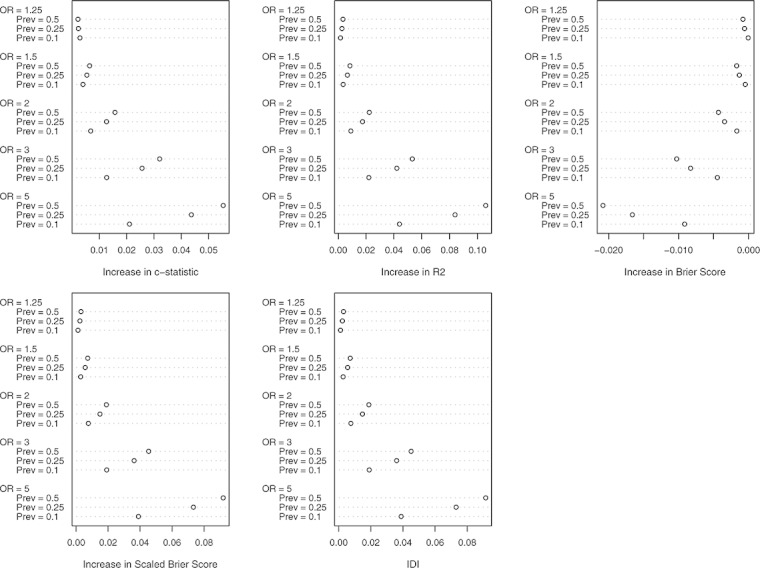
Effect of odds ratio and prevalence of risk factor on increase in model accuracy.

As noted previously, when the risk factor was dichotomous, improvements in model accuracy were proportional to the prevalence of the risk factor and to the odds ratio relating the risk factor to the binary outcome. We explored the nature of this relationship further in the setting in which the dichotomous risk factor was independent of the continuous covariate. Using the results from the 3120 scenarios in this setting, we used an analysis of variance model to regress the improvement in model accuracy on the following three variables: the accuracy of the model with only the single continuous covariate (1 df), the prevalence of the dichotomous risk factor (2 df), and the odds ratio of the dichotomous risk factor (4 df), along with all two-way interactions. Using the fitted model, we then estimated the change in model accuracy for each combination of prevalence and odds ratio when the accuracy of the univariable model was set to the median value across the 3120 scenarios. We report the results in the dot charts in Figure 8. The most striking observation is the interaction between the prevalence of the risk factor and the odds ratio relating it to the outcome. When the odds ratio was low ( OR = 1.25), increasing the prevalence of the risk factor had a negligible impact on model accuracy. However, as the odds ratio increased, the effect of increasing prevalence was amplified. When the odds ratio was large ( OR = 5), then the effect of increasing prevalence on model accuracy was strong.

## 5 Case study

We briefly provide a case study to examine the change in measures of model performance when a novel risk factor is added to an existing logistic regression models.

### 5.1 Data

The EFFECT Study is an initiative intended to improve the quality of care for patients with cardiovascular disease in Ontario 13,14. During the first phase of the study, detailed clinical data on patients hospitalized with heart failure between April 1, 1999 and March 31, 2001, at 103 hospitals in Ontario, Canada, were obtained by retrospective chart review. Data on patient demographics, vital signs and physical examination at presentation, medical history, and results of laboratory tests were collected for this sample. Subjects with missing data on continuous baseline covariates necessary to estimate the risk scores were excluded from the current case study. The case study used 8635 patients who were hospitalized for acute heart failure.

### 5.2 Methods

We considered two models for predicting death within 30 days of hospital admission. The first model used patient age as the only predictor of mortality. The second model used the EFFECT-HF mortality prediction model 1. The EFFECT-HF mortality prediction model is a point-based scoring system for predicting the risk of 30-day and 1-year mortality. It uses the following patient characteristics to determine a score for predicting short-term mortality: age, respiratory rate, systolic blood pressure, urea nitrogen, sodium concentration, cerebrovascular disease, dementia, chronic obstructive pulmonary disease, hepatic cirrhosis, and cancer. The novel risk factor that we considered was whether the patient had documented evidence of frailty in the medical record, defined as occurring if the physician used the term ‘frail’ in the patient's medical record.

We fit four separate logistic regression models to predict 30-day mortality. The regression model used the following sets of predictor variables: (i) age as the sole predictor; (ii) age and frailty as two predictor variables; (iii) the EFFECT-HF risk score as the sole predictor; and (iv) the EFFECT-HF risk score and frailty as two predictor variables. For each logistic regression model, we computed the following performance measures: c-statistic, generalized *R*^2^, discrimination slope, Brier score, and the scaled Brier score. We determined the change in each measure of model performance when an indicator variable denoting the presence of frailty was added to each of the univariate models.

### 5.3 Results

The c-statistics of the logistic regression models with either age or the EFFECT-HF score as the only predictor variables were 0.636 and 0.753, respectively (Table 1). Both the absolute and relative increase in the c-statistic were greater when an indicator variable denoting the presence of frailty was added to the model with age than when the same marker was added to the model with the EFFECT-HF score (absolute change: 0.005 vs. 0.001). Similarly, there was a greater improvement in the other performance measures (change in the model *R*^2^, (scaled) Brier score, and the change in discrimination slope (IDI)).

**Table 1:** Measures of performance for different logistic regression models in the EFFECT-HF sample of patients hospitalized with acute heart failure.

Performance measure	Logistic regression model				
Age	Age + frailty	EFFECT-HF score	EFFECT-HF score + frailty
c-statistic	0.636	0.641	0.753	0.754
*R*^2^ index	0.046	0.050	0.162	0.163
Brier score	0.0933	0.0931	0.0863	0.0862
Scaled Brier score	0.024	0.026	0.098	0.098
Discrimination slope	0.023	0.026	0.099	0.100

The relative change in model performance due to the addition of frailty to the model that contained age as the sole predictor variable was, in order of increasing relative change: Brier score (0.2% increase), c-statistic (0.8% increase), scaled Brier score (8.3% increase), *R*^2^ (8.7% increase), and the discrimination slope (13.0%).

In summary, we observed phenomena in the case study similar to the results of our series of Monte Carlo simulations. In particular, greater improvements in model accuracy due to the inclusion of a binary risk factor are possible when the initial model has lower performance compared with when the initial model has higher performance.

## 6 Discussion

The improvement in model accuracy due to the addition of either a binary or a continuous risk factor to an existing logistic regression model was inversely proportional to the accuracy of the initial model. For each measure of model accuracy, the improvement in accuracy was proportional to the odds ratio relating the risk factor to the outcome. Furthermore, when the risk factor was dichotomous, the improvement in model accuracy was proportional to the prevalence of the dichotomous risk factor. For a given measure of model accuracy, the improvement in model accuracy due to the inclusion of the risk factor tended to decrease as the correlation between the risk factor and the continuous predictor variable increased. We observed the greatest increase in model accuracy when the risk factor and the predictor variable were uncorrelated with one another. Finally, when the risk factor was dichotomous, we observed a synergistic effect of the odds ratio and the prevalence of the dichotomous risk factor on the increase in model accuracy. There are important implications of these observations for researchers examining the ability of novel risk factors to predict outcomes. First, only minor to modest improvements in predictive accuracy are feasible when incorporating a new risk factor when the pre-existing regression model already has high accuracy 15. Conversely, greater improvements are possible when the pre-existing model has low accuracy. Second, greater improvements in accuracy will be observed when a dichotomous risk factor with higher prevalence is added to a regression model compared with when a dichotomous risk factor with lower prevalence is added. Third, greater improvements in accuracy are possible when the odds ratio relating the risk factor to the outcome is larger 16. Finally, greater improvements in accuracy are possible when the risk factor is uncorrelated with the existing predictor compared with when the risk factor has a positive correlation with the existing predictor variable.

Tzoulaki *et al.* reviewed articles examining the improvements in predicting cardiovascular events when an additional risk factor was added to the Framingham risk score (FRS) 2009. In studies that reported the increase in c-statistic when an additional risk factor was added to the FRS, they observed that the increase in c-statistic was inversely proportional to the c-statistic of the regression model that only included the FRS. They write that ‘this may be due to chance (regression to the mean), genuine differences in study characteristics, or a conglomerate of diverse biases that decrease the predictive performance of the FRS’ (p. 2349) and that ‘studies without these methodological flaws did not show any substantial improvements in AUC’ (p. 2450). Our simulations demonstrated that the inverse relationship between the increase in the c-statistic and the c-statistic of the model with only the FRS was likely not due to chance alone, to bias, or to regression to the mean. Instead, this observation appears to be an underlying property of the c-statistic and other measures of model accuracy: given the same risk factor or marker, greater improvements in predictive accuracy are possible in settings in which the underlying regression model has lower discrimination compared with settings in which it has higher discrimination.

A limitation of the current study is that we did not consider recently proposed measures that consider the ability of risk factors to improve classification accuracy, nor did we consider decision analytic measures that incorporate the relative costs of false-positive and false-negative decisions 6,18–21. In the current study, we focused our attention on predicting the risk or probability of an event, rather than on categorizing patients into different states. Examining the effects of adding risk factors on the accuracy of classifications is beyond the scope of the current study. Another limitation is that we only considered the situation in which a risk factor (either dichotomous or continuous) was added to a logistic regression model that consisted of either a single continuous predictor variable or of a continuous predictor variable and a dichotomous predictor variable. We consider it likely that our results generalize to situations of a reference model with a mixture of more than two categorical and continuous predictors. Finally, we focused on odds ratios in the range of 1.25 to 5, whereas many genetic markers may have smaller effects, for example, around 1.1 to 1.25. These genetic markers may then be summarized in gene scores or risk profiles 22. Again, on the basis of our results with an odds ratio of 1.25, we consider it likely that our results regarding the behavior of these performance measures generalize to these situations of limited predictive genetic effects.

In the current study, we restricted our attention to predicting the probability of the occurrence of a binary outcome and did not consider settings with survival or time-to-event outcomes. Several authors have proposed different extensions of the c-statistic or the AUC for use with time-to-event outcomes in the presence of censoring 23–27. Chambless *et al.* have proposed extensions of the *R*^2^ and the IDI for use with survival outcomes in the presence of censoring 2011. A comprehensive examination of the sensitivity of these different measures of predictive accuracy for survival outcomes to the inclusion of novel risk factors or markers merits examination in a separate paper. However, we would expect similar findings for these extensions of measures of model accuracy when applied to Cox regression models with binary or continuous markers. In particular, we would expect that for a given measure of accuracy, the improvement in model accuracy would be proportional to the hazard ratio relating the risk factor to the outcome. Furthermore, when the risk factor is dichotomous, we would anticipate that the improvement in model accuracy would be proportional to the prevalence of the dichotomous risk factor.

In conclusion, improvements in accuracy of a logistic regression model due to the inclusion of a novel risk factor or marker need to be interpreted in their context, specifically the accuracy of the model with the risk factor omitted.

## References

[b1] Lee DS, Austin PC, Rouleau JL, Liu PP, Naimark D, Tu JV (2003). Predicting mortality among patients hospitalized for heart failure: derivation and validation of a clinical model. Journal of the American Medical Association.

[b2] Lee KL, Woodlief LH, Topol EJ, Weaver WD, Betriu A, Col J, Simoons M, Aylward P, Van de WF, Califf RM (1995). Predictors of 30-day mortality in the era of reperfusion for acute myocardial infarction. Results from an international trial of 41,021 patients. GUSTO-I Investigators. Circulation.

[b3] Roozenbeek B, Maas AI, Lingsma HF, Butcher I, Lu J, Marmarou A, McHugh GS, Weir J, Murray GD, Steyerberg EW (2009). IMPACT Study Group. Baseline characteristics and statistical power in randomized controlled trials: selection, prognostic targeting, or covariate adjustment?. Critical Care Medicine.

[b4] Steyerberg EW (2009). Clinical Prediction Models: A Practical Approach to Development, Validation, and Updating.

[b5] Steyerberg EW, Vickers AJ, Cook NR, Gerds T, Gonen M, Obuchowski N, Pencina MJ, Kattan MW (2010). Assessing the performance of prediction models: a framework for traditional and novel measures. Epidemiology.

[b6] Pencina MJ, D'Agostino Sr RB, D'Agostino Jr RB, Vasan RS (2008). Evaluating the added predictive ability of a new marker: from area under the ROC curve to reclassification and beyond. Statistics in Medicine.

[b7] Harrell FEJ (2001). Regression Modeling Strategies.

[b8] Cook NR (2007). Use and misuse of the receiver operating characteristic curve in risk prediction. Circulation.

[b9] Nagelkerke NJD (1991). A note on a general definition of the coefficient of determination. Biometrika.

[b10] Cragg JG, Uhler R (1970). The demand for automobiles. Canadian Journal of Economics.

[b11] Pepe MS, Feng Z, Gu JW, Pencina MJ (2008). Comments on ‘Evaluating the added predictive ability of a new marker: From area under the ROC curve to reclassification and beyond’. Statistics in Medicine.

[b12] R Core Development Team (2005). R: A Language and Environment for Statistical Computing.

[b13] Tu JV, Donovan LR, Lee DS, Wang JT, Austin PC, Alter DA, Ko DT (2009). Effectiveness of public report cards for improving the quality of cardiac care: the EFFECT study: a randomized trial. Journal of the American Medical Association.

[b14] Tu JV, Donovan LR, Lee DS, Austin PC, Ko DT, Wang JT, Newman AM (2004). Quality of Cardiac Care in Ontario.

[b15] Pepe MS, Janes H, Longton G, Leisenring W, Newcomb P (2004). Limitations of the odds ratio in gauging the performance of a diagnostic, prognostic, or screening marker. American Journal of Epidemiology.

[b16] Janssens AC, Moonesinghe R, Yang Q, Steyerberg EW, van D, Khoury MJ (2007). The impact of genotype frequencies on the clinical validity of genomic profiling for predicting common chronic diseases. Genetics in Medicine.

[b17] Tzoulaki I, Liberopoulos G, Ioannidis JP (2009). Assessment of claims of improved prediction beyond the Framingham risk score. Journal of the American Medical Association.

[b18] Pencina MJ, D'Agostino Sr RB, Steyerberg EW (2011). Extensions of net reclassification improvement calculations to measure usefulness of new biomarkers. Statistics in Medicine.

[b19] Vickers AJ, Elkin EB (2006). Decision curve analysis: a novel method for evaluating prediction models. Medical Decision Making.

[b20] Baker SG (2009). Putting risk prediction in perspective: relative utility curves. Journal of the National Cancer Institute.

[b21] Vickers AJ, Cronin AM, Begg CB (2011). One statistical test is sufficient for assessing new predictive markers. BMC Medical Research Methodology.

[b22] van der Net JB, Janssens AC, Sijbrands EJ, Steyerberg EW (2009). Value of genetic profiling for the prediction of coronary heart disease. American Heart Journal.

[b23] Harrell Jr FE, Lee KL, Mark DB (1996). Multivariable prognostic models: issues in developing models, evaluating assumptions and adequacy, and measuring and reducing errors. Statistics in Medicine.

[b24] Pencina MJ, D'Agostino Sr RB, Song L (2012). Quantifying discrimination of Framingham risk functions with different survival C statistics. Statistics in Medicine.

[b25] Pencina MJ, D'Agostino RB (2004). Overall C as a measure of discrimination in survival analysis: model specific population value and confidence interval estimation. Statistics in Medicine.

[b26] Uno H, Cai T, Pencina MJ, D'Agostino RB, Wei LJ (2011). On the C-statistics for evaluating overall adequacy of risk prediction procedures with censored survival data. Statistics in Medicine.

[b27] Chambless LE, Diao G (2006). Estimation of time-dependent area under the ROC curve for long-term risk prediction. Statistics in Medicine.

[b28] Chambless LE, Cummiskey CP, Cui G (2011). Several methods to assess improvement in risk prediction models: extension to survival analysis. Statistics in Medicine.

